# The Flavin Reductase MsuE Is a Novel Nitroreductase that Can Efficiently Activate Two Promising Next-Generation Prodrugs for Gene-Directed Enzyme Prodrug Therapy

**DOI:** 10.3390/cancers5030985

**Published:** 2013-08-08

**Authors:** Laura K. Green, Mathew A. Storey, Elsie M. Williams, Adam V. Patterson, Jeff B. Smaill, Janine N. Copp, David F. Ackerley

**Affiliations:** 1 School of Biological Sciences, Victoria University of Wellington, Kelburn Parade, Wellington 6140, New Zealand; 2 Victoria University Centre for Biodiscovery, School of Biological Sciences, Victoria University of Wellington, Wellington 6140, New Zealand; 3 Maurice Wilkins Centre for Molecular Biodiscovery, School of Biological Sciences, University of Auckland, Auckland 1142, New Zealand; 4 Auckland Cancer Society Research Centre, University of Auckland, Grafton, Auckland 1142, New Zealand

**Keywords:** gene therapy, GDEPT, nitroaromatic prodrug, nitroreductase, CB1954, PR-104A, Nitro-CBI-DEI, SOS chromotest

## Abstract

Bacterial nitroreductase enzymes that can efficiently catalyse the oxygen-independent reduction of prodrugs originally developed to target tumour hypoxia offer great potential for expanding the therapeutic range of these molecules to aerobic tumour regions, via the emerging cancer strategy of gene-directed enzyme prodrug therapy (GDEPT). Two promising hypoxia prodrugs for GDEPT are the dinitrobenzamide mustard PR-104A, and the nitrochloromethylbenzindoline prodrug nitro-CBI-DEI. We describe here use of a nitro-quenched fluorogenic probe to identify MsuE from *Pseudomonas aeruginosa* as a novel nitroreductase candidate for GDEPT. In SOS and bacteria-delivered enzyme prodrug cytotoxicity assays MsuE was less effective at activating CB1954 (a first-generation GDEPT prodrug) than the “gold standard” nitroreductases NfsA and NfsB from *Escherichia coli*. However, MsuE exhibited comparable levels of activity with PR-104A and nitro-CBI-DEI, and is the first nitroreductase outside of the NfsA and NfsB enzyme families to do so. These *in vitro* findings suggest that MsuE is worthy of further evaluation in *in vivo* models of GDEPT.

## 1. Introduction

In gene-directed enzyme prodrug therapy (GDEPT), tumour tropic vectors are “armed” with a transgene that encodes a prodrug converting enzyme, enabling them to selectively sensitise tumour cells to that prodrug. A historical limitation of cancer gene therapies is their inability to transfect more than a small minority of cancerous cells [1,2]. In GDEPT, low transfection rates can be countered to a substantial degree by: (I) using a prodrug that exerts a strong bystander effect (*i.e.*, the active metabolite(s) can efficiently penetrate and kill untransfected cells proximal to the site of activation); and (II) developing more efficient enzymes to achieve maximal prodrug conversion.

Therapeutic genes employed in GDEPT are typically non-human in origin, to minimise the potential for prodrug activation by endogenous enzymes in healthy tissue. Two of the most widely studied genes for GDEPT are herpes simplex virus thymidine kinase, which was evaluated in phase III clinical trials in combination with the nucleoside analogue prodrug ganciclovir [3]; and cytosine deaminase from *Escherichia coli*, which has undergone phase I trial in combination with another nucleoside analogue prodrug, 5-fluorocytosine [4]. These systems enjoy the substantial benefit that their prodrugs have each achieved independent clinical utility; but they also suffer from the limitations that only actively dividing cells are targeted, that their activated metabolites inhibit replication of viral vectors, and their cell-to-cell bystander effects are enhanced by functional gap junctions, which many tumour cells lack [1,2,5].

A third enzyme-prodrug combination that has undergone clinical evaluation for GDEPT is *E. coli* NfsB (NfsB_Ec) in combination with CB1954 [5-(aziridin-1-yl)-2,4-dinitrobenzamide] [6,7]. The simultaneous 2-electron reduction of CB1954 by NfsB_Ec generates activated metabolites that can freely diffuse across cell membranes, forming adducts and DNA crosslinks, and inducing apoptosis in both replicating and quiescent tumour cells [8,9]. Nonetheless, results of the clinical trials were somewhat equivocal—while there was evidence for a slight anti-tumour effect, CB1954 was also discovered to exhibit dose-limiting hepatotoxicity, and the administered dose yielded a serum concentration of prodrug that was approximately two orders of magnitude lower than the *K_m_* of NfsB_Ec [6,7,10]. In our on-going research we have sought to improve the efficacy of CB1954 GDEPT through identification and engineering of nitroreductases that exhibit superior activity over NfsB_Ec at concentrations of prodrug that are attainable *in vivo* [11,12,13,14]. However, it is also known that CB1954 exhibits a relatively modest bystander effect relative to other nitroaromatic prodrugs with potential utility in GDEPT [15]. Thus, we have sought in parallel to identify superior prodrug substrates for nitroreductase GDEPT.

Two particularly promising next-generation prodrugs for nitroreductase GDEPT are nitro-CBI-DEI (nitro-CBI-5-[(dimethylamino)ethoxy]indole [16,17]) and PR-104A (2-(2-bromoethyl)-2-{[(2-hydroxyethyl)amino]carbonyl}-4,6-dinitroanilino)ethyl methanesulfonate [18]), each originally developed to be activated by tumour hypoxia. The rationale underpinning design of these nitro-triggered hypoxia prodrugs is that human oxidoreductases reduce these molecules via single-electron transfer, forming an initial nitro radical anion that molecular oxygen in healthy tissue rapidly back-oxidises, restoring the prodrug form in a “futile cycle” [19]. In contrast, we have shown that multiple bacterial nitroreductases from different enzyme families have the ability to reduce these molecules via oxygen-independent concerted two electron steps, yielding end metabolites that are highly cytotoxic and also exhibit substantial bystander effects [14,17].

Previously, we have identified five different oxygen-independent bacterial enzyme families (NfsA, NfsB, NemA, AzoR and MdaB, each named after the orthologous enzyme from *E. coli*) as containing nitroreductases that can activate at least one of CB1954, PR-104A and nitro-CBI-DEI [14,17]. Of these, NfsA and NfsB family members consistently exhibit substantially greater activity with all three prodrugs than any of the other nitroreductase families. Two other enzyme families have been identified as having CB1954 reductase activity, YieF [20] and YwrO [9,21]; however to date we have not found any members of these families to exhibit measurable levels of activity with either PR-104A or nitro-CBI-DEI. Here we describe identification of MsuE from *Pseudomonas aeruginosa* as a novel nitroreductase, the first report of a non-NfsA or NfsB type enzyme to exhibit comparable levels of activity with these next-generation prodrugs for GDEPT.

## 2. Results and Discussion

### 2.1. Identification of P. aeruginosa MsuE as a Nitroreductase Enzyme

**Figure 1:**
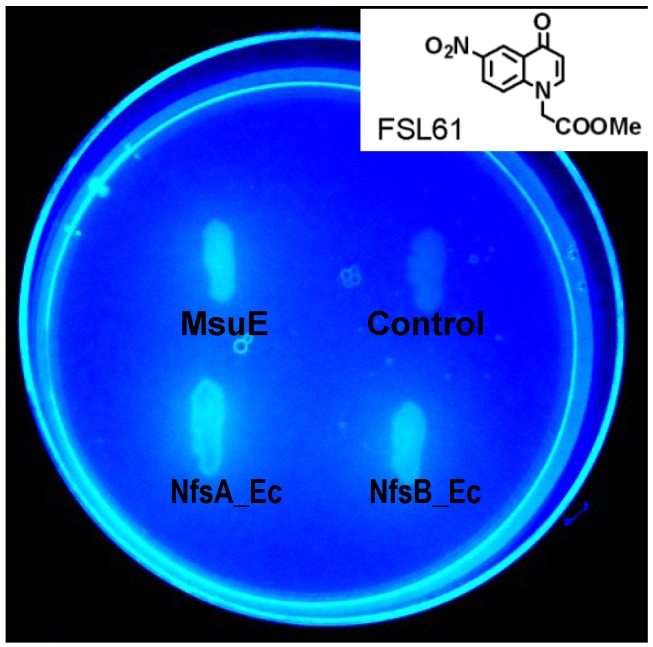
Nitroreductase-dependent activation of the fluorogenic probe FSL 61. *E. coli* SOS-R2 strains over-expressing NfsA_Ec, NfsB_Ec or MsuE as indicated, or containing an empty pUCX plasmid (“Control”), were streaked on an LB agar plate amended with 100 µg/mL ampicillin and 25 µM of the nitro-quenched fluorophore FSL 61 (structure inset). The image was taken using a hand-held Panasonic DMC-LS5 digital camera, with the agar plate underlit by a broad-range ultra-violet transilluminator.

In our ongoing attempts to identify the most promising nitroreductases for GDEPT we have conducted a systematic evaluation of NfsA, NfsB, NemA, AzoR and MdaB family members from (to date) 25 different bacterial species [14,22]. We have augmented this family-based approach with individual evaluations of unrelated candidates, based on published structural and functional characteristics consistent with possible nitroreductase activity (*i.e.*, homodimeric, NAD(P)H-consuming, flavin-cofactor-dependent, cytoplasmic oxidoreductases [14,23]). One such candidate was MsuE from *P. aeruginosa*, previously characterised as an NADH-dependent flavin mononucleotide (FMN) reductase that provides reduced FMN to power desulfonation of alkanesulfonates [24,25]. In an initial rapid qualitative test for nitroreductase activity, employing the nitro-quenched fluorophore FSL 61 as a generic nitroreductase substrate (as per [14]), a nitroreductase gene knockout strain of *E. coli* over-expressing MsuE from plasmid pUCX exhibited comparable levels of fluorescence to isogenic strains over-expressing *E. coli* NfsA (NfsA_Ec) or NfsB_Ec (Figure 1). In contrast, an empty-plasmid control strain was unable to activate the fluorophore (Figure 1). SDS-PAGE analysis coupled with scanning densitometry indicated that MsuE was expressed from pUCX in *E. coli* at approximately 35% the level of NfsA_Ec and 38% the level of NfsB_Ec.

### 2.2. MsuE Is Genetically Distinct from Previously Identified Bacterial Nitroreductases

**Figure 2:**
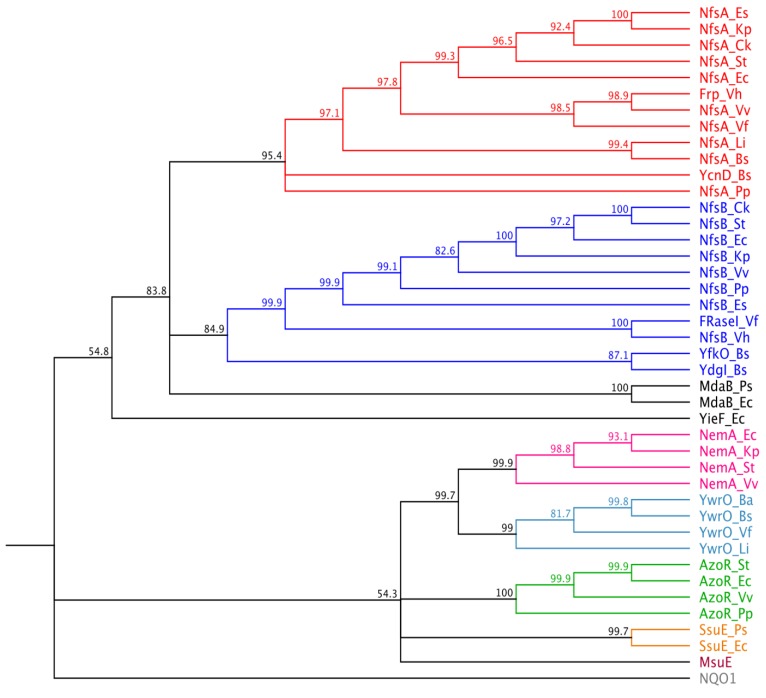
Phylogenetic analysis of MsuE and other experimentally validated bacterial nitroreductase families. MsuE (dark red) and two SsuE orthologues from *E. coli* and *Pseudomonas syringae pv.* tomato DC3000 (orange) were genetically aligned against members of the NfsA (red), NfsB (dark blue), MdaB (black), YieF (black), NemA (pink), YwrO (light blue) and AzoR (green) nitroreductase families, and a human NQO1 out-group (grey), using the online tool ClustalW2. With the exception of the *msuE* and *ssuE* genes, nitroreductase sequences are as previously presented by Prosser *et al.* [14]. Following the nomenclature of that paper, the two letter suffix following the enzyme name indicates the bacterial strain of origin: Ba, *B. amyloliquefaciens*; Bs, *Bacillus subtilis*; Ck, *Citrobacter koseri*; Es, *Enterobacter sakazakii*; Ec, *Escherichia coli*; Kp, *Klebsiella pneumonia*; Li, *Listeria innocua*; Pa, *Pseudomonas aeruginosa*; Pp, *Pseudomonas putida*; Ps, *Pseudomonas syringae*; St, *Salmonella typhi*; Vf, *Vibrio fischeri*; Vh, *Vibrio harveyi*; Vv, *Vibrio vulnificus*. The phylogenetic tree was constructed using the online tool MrBayes 3.2.1 [27] and bootstrap confidence values are indicated at major branchpoints. The final figure was generated using Geneious version 6.1 [28].

When aligned against members of all other previously identified bacterial nitroreductase families using ClustalW2, with an out-group of human NAD(P)H quinone oxidoreductase 1 (NQO1, also known as DT-diaphorase [26]), the *msuE* gene formed a distinct clade (Figure 2). At an amino acid level, MsuE and its *E. coli* orthologue SsuE share nearly 30% identity with one other, but each less than 15% identity with members of the AzoR family, their nearest neighbours in the ClustalW2 alignment. These observations suggest that MsuE belongs to a separate enzyme family from all other previously identified nitroreductases. Of particular note, our alignments revealed two highly conserved regions in each of the NfsA and NfsB families (NfsA: AAES[L/M/Q]G[F/L]GxxIG[A/G][I/L/M/V]R and KPR[L/M]Pxx[A/L/M/V][I/L/M/V]xHE[E/N]; and NfsB: [Q/E]PW[H/Q/R]F[F/I/L/V]V[A/I/V] and D[A/S/T]xP[I/M]EG[F/I/V][D/H/Q]). None of these conserved sequences are present in MsuE.

### 2.3. MsuE Is Able to Activate Promising Next-Generation GDEPT Prodrugs

**Figure 3:**
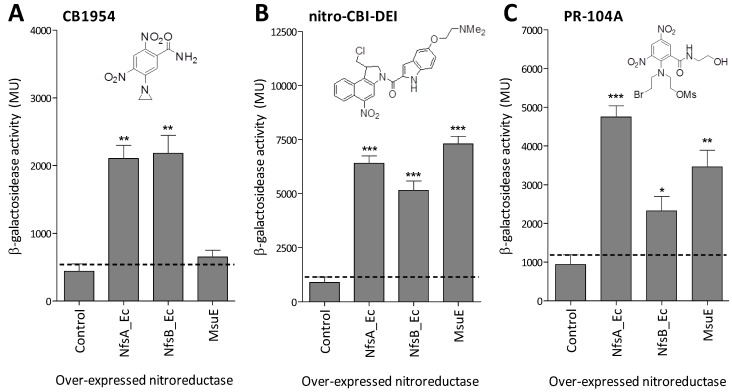
Fold-induction of SOS response in SOS-R2 strains over-expressing NfsA_Ec, NfsB_Ec or MsuE upon challenge with CB1954, nitro-CBI-DEI or PR-104A. SOS-R2 reporter strains over-expressing *nfsA_Ec*, *nfsB_Ec* or *msuE*, or containing an empty pUCX plasmid control, were incubated for 3 h in the presence of (**A**) 20 µM CB1954; (**B**) 5 µM nitro-CBI-DEI; or (**C**) 40 µM PR-104A (structures inset); after which relative induction of the SOS response in each strain was measured by β-galactosidase assay as previously described [13]. Data are the mean Miller units from three independent experiments, each performed in duplicate; and error bars are ± 1 standard deviation. The black dotted line indicates the upper error limit of basal SOS activity in the empty plasmid control. * indicates *p* < 0.05, ** indicates *p* < 0.005, *** indicates *p* < 0.001 (one-way ANOVA with Dunnett comparison of test to control).

We next conducted a preliminary test of whether MsuE was able to catalyse bioreductive activation of the prodrugs CB1954, PR-104A or nitro-CBI-DEI at comparable levels to the prototypical nitroreductases NfsA_Ec and NfsB_Ec. To achieve this, each enzyme was individually over-expressed in the *E. coli* reporter strain SOS-R2, which lacks endogenous nitroreductases and contains a *lacZ* gene under control of the SOS (DNA damage responsive) promoter *sfiA*. We have previously demonstrated that this reporter system provides a quantitative measure of genotoxic prodrug activation by over-expressed nitroreductase candidates [14]. Upon challenge with 20 µM CB1954, only NfsA_Ec and NfsB_Ec induced a significant SOS response relative to the empty-plasmid control (*p* < 0.005; Figure 3A). However, following challenge with 5 µM nitro-CBI-DEI or 40 µM PR-104A, all three enzymes generated a significant SOS response relative to the control (*p* < 0.05; Figure 3B,C). With nitro-CBI-DEI, all three enzymes had comparable activity in this system; while with PR-104A, MsuE was more active than NfsB_Ec, but less active than NfsA_Ec.

### 2.4. MsuE Is Able to Sensitise Human Colon Carcinoma Cells to Bioreductive Prodrugs

**Figure 4:**
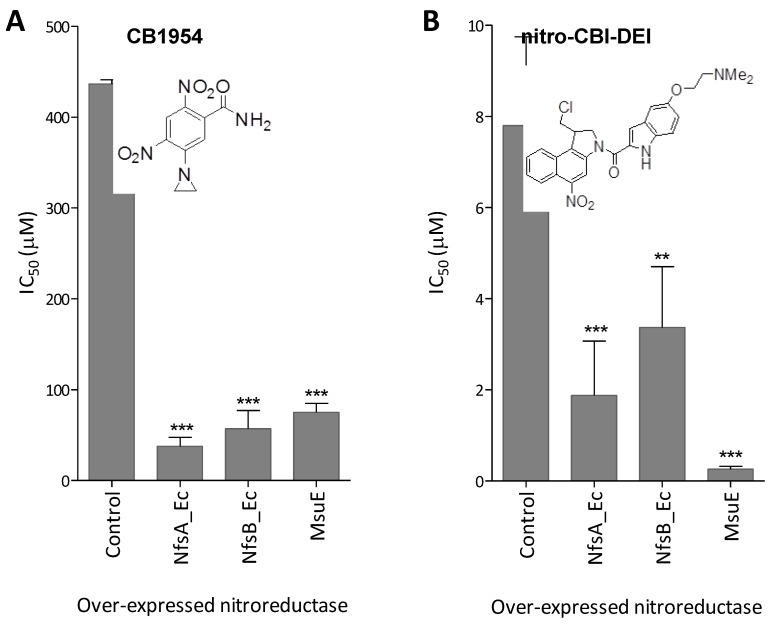
Bacteria-delivered enzyme prodrug cytotoxicity assay. Calculated IC_50_ of HCT-116 cells post-incubation with *E. coli 6KO* cells (over-expressing either NfsA_Ec, NfsB_Ec, MsuE, or an empty pUCX control) across a 2-fold dilution series of (**A**) CB1954 (25 µM to 400 µM); or (**B**) nitro-CBI-DEI (0.06 µM to 15 µM). Percentage cell survival at each prodrug concentration was calculated relative to an unchallenged control by CellTiter 96^®^ AQ_ueous_ One Solution Cell Proliferation Assay (Promega, Madison, WI, USA). Data are the mean of three independent experiments, each performed in duplicate; and error bars are ±1 standard deviation. ** indicates *p* < 0.005 and *** *p* < 0.001 by one-way ANOVA with Dunnett comparison of test to control.

In an effort to measure the relative abilities of MsuE, NfsA_Ec and NfsB_Ec to sensitise human colon carcinoma (HCT-116) cells to each prodrug we employed a bacteria-delivered enzyme cytotoxicity assay as previously described [13]. *E. coli* strains individually over-expressing each enzyme or an empty plasmid control were incubated in co-culture with replicate monolayers of HCT-116 cells, across a range of concentrations of each prodrug, after which IC_50_ values were measured (*i.e.*, the concentration of prodrug required to reduce the viability of the HCT-116 cells to 50% relative to the no-prodrug control). In this assay system, independent experiments using CB1954 or nitro-CBI-DEI gave highly reproducible results for all strains. Despite MsuE exhibiting only limited activity with CB1954 in SOS assays (Section 2.3), HCT-116 cells co-cultured with the *E. coli* strain over-expressing this nitroreductase were 5.8-fold more sensitive to CB1954 than cells co-cultured with the empty plasmid control strain (Figure 4A). Consistent with the SOS assays, NfsA_Ec and NfsB_Ec were more effective than MsuE in sensitising HCT-116 cells to CB1954 (Figure 4A). With nitro-CBI-DEI however, MsuE was the most effective nitroreductase in sensitising HCT-116 cells to the administered prodrug (Figure 4B). The IC_50_ of 260 ± 40 nM measured in this assay system is comparable to that previously described for the most active nitro-CBI-DEI reductase yet to be identified (*P. aeruginosa* NfsB, 130 nM [17]); and substantially (although not quite significantly; *p* < 0.06) less than that measured for the best *E. coli* nitroreductase, NfsA_Ec (1.9 ± 1.2 µM; Figure 4B). Surprisingly, PR-104A was unable to exert a comparable effect in this assay, possibly due to the relatively reactive nature of the metabolites; irrespective of the conditions employed, concentration-dependent IC_50_ curves could not be obtained for any of the *E. coli* strains. Overall, based on the results of the SOS and bacteria-delivered enzyme cytotoxicity assays, MsuE appears to be a promising candidate for GDEPT, offering potential to repurpose next-generation prodrugs that have been independently developed as hypoxia activated therapeutics.

## 3. Experimental

### 3.1. Chemicals, Bacterial Strains, Plasmids and Bacterial Culture Conditions

Chemicals (including CB1954), reagents and growth media were obtained from Sigma-Aldrich (St. Louis, MO, USA) unless otherwise stated. PR-104A and the fluorogenic probe FSL61 were synthesised in-house at the Auckland Cancer Society Research Centre (ACSRC). Nitro-CBI-DEI was a generous gift from Dr Moana Tercel at the ACSRC.

The *E. coli* SOS reporter strain SOS-R2, gene deleted in four endogenous nitroreductases (*nfsA nfsB nemA azoR*) and the efflux regulator *tolC* (construction described in [14]) was used as the default host strain for all experiments involving nitroreductase gene over-expression unless otherwise stated. NfsA_Ec and NfsB_Ec were over-expressed from plasmid pUCX, an expression plasmid derived from pUC19, as previously described [11]. The *msuE* gene was PCR-amplified from *P. aeruginosa* PAO1 genomic DNA using oligonucleotide primers purchased from Integrated DNA Technologies (Coralville, IA, USA): *msuE_for* CCCCATATGACCAGCCCCTTCAAA and *msuE_rev* CCCGTCGACTCAGGCGATCTTCAACGG; and cloned into pUCX using the *Nde*I and *Sal*I restriction sites (underlined). Successful error-free PCR amplification and cloning was verified by sequencing (Macrogen Inc., Seoul, Korea). For bacteria-delivered enzyme prodrug cytotoxicity assays, all nitroreductases were expressed from pUCX in an *nfsA nfsB azoR mdaB yieF ycaK* (“*Δ6KO*”) deletion strain of *E. coli* W3110 (the intact *tolC* gene in this strain promotes efflux of activated metabolites relative to SOS-R2). Luria-Bertani (LB) liquid or solid (1.5% agar) media containing 100 µg/mL ampicillin was used for propagation of *E. coli* strains harboring pUCX plasmids. Solid LB media was amended with 25 µM FSL 61 for fluorescence screening.

### 3.2. Bioinformatic Analyses

The MsuE, SsuE_Ec and SsuE_Ps protein and gene sequences were downloaded from PubMed databases [29] (GenBank protein accession numbers AAG05745.1, BAA35692.1 and AAO56926.1, respectively). The multiple sequence alignment was conducted using ClustalW2 [30]. The phylogenetic tree in Figure 2 was reconstructed from this alignment using MrBayes 3.2.1 [27]. All other sequence analyses were performed using Geneious version 6.1 [28].

### 3.3. SOS Assays

Individual wells of a 96-well microtitre plate containing 100 µL LB amended with 100 µg/mL ampicillin and 0.4% (w/v) glucose were inoculated in duplicate with SOS-R2 strains over-expressing NfsA_Ec, NfsB_Ec or MsuE, or else containing a pUCX empty plasmid control, and incubated overnight at 30 °C with shaking at 200 rpm. The next morning, 15 µL from each well was sub-cultured into 200 µL of fresh medium (LB supplemented 100 µg/mL ampicillin, 0.42% (w/v) glucose and 50 µM IPTG) in a fresh 96-well microtitre plate. The fresh plates were then incubated at 30 °C with shaking at 200 rpm for 3.5 h for induction of nitroreductase expression. Cultures were then split (100 µL each) into two fresh 96 well microtitre plates. Prodrug challenge was initiated in one of the replicate plates by addition of 100 µL fresh assay media containing prodrug at 2× the desired final concentration and a DMSO concentration of 1% (v/v). The remaining microtitre plate (unchallenged control) was treated identically except that prodrug was omitted from the media. After 3 h incubation at 30 °C with shaking (200 rpm), turbidity (OD_600_) was recorded and then 10 µL of each culture was added to 140 µL ZOB buffer (see below) for analysis of b-galactosidase activity in fresh 96-well microtitre plates. Reactions were then incubated at 37 °C for 25 min, at which point the assay was terminated by addition of 50 µL of 1 M Na_2_CO_3_ and absorbance at 420 nm and 550 nm were recorded. Miller units of ONPG were calculated using a modified Miller equation as previously described [11]. The ZOB buffer used in this study was a 9:4:1 (v/v) mixture of 50 mM sodium phosphate buffer (pH 7.0), Z buffer and T base, respectively, where Z buffer is 74 mM NaH_2_PO_4_, 126 mM Na_2_HPO_4_, 2 mM MgSO_4_, 0.4 mM MnSO_4_, 400 mg/L hexadecyltrimethylammonium bromide (CTAB), 200 mg/L sodium deoxycholate and 174 mM (12.2 µL/mL) β-mercaptoethanol (the latter added immediately prior to use); and T base is 80 mM K_2_HPO_4_, 44 mM KH_2_PO_4_, 1 g/L trisodium citrate, 15.1 mM (NH_4_)_2_SO_4_, and 8 g/L ONPG (the latter added immediately prior to use).

### 3.4. Bacteria-Delivered Enzyme Prodrug Cytotoxicity Assays

HCT-116 colorectal carcinoma cells (American Type Culture Collection, Manassas, VA, USA) were seeded into 96-well microtitre plate wells at 5 × 10^3^ per 100 μL of cell culture media (CMEM supplemented with 5% foetal calf serum and 10 mM HEPES (Gibco, Grand Island, NY, USA) and incubated at 37 °C, 5% CO_2_ for 48 h. In separate 96-well microtitre plates, *E. coli Δ6KO* strains over-expressing each nitroreductase from plasmid pUCX, or else transformed with an empty pUCX control, were individually inoculated into 150 μL LB amended with 100 µg/mL ampicillin and 0.4% (w/v) glucose, then incubated for 16 h at 30 °C with shaking at 200 rpm. The next day, 15 μL from each overnight culture were transferred to fresh 96-well microtitre plate wells containing 200 μL cell culture media amended with 100 µg/mL ampicillin, 0.05 mM IPTG and 0.4% (w/v) glucose (CMEM-AIG), and incubated at 30 °C with shaking at 200 rpm for 3 h. During this incubation period, a 2-fold dilution series of CB1954 (from 400 µM to 25 µM, plus no-prodrug control) and nitro-CBI-DEI (from 15 µM to 0.06 µM, plus no-prodrug control) were prepared in CMEM-AIG. The 3 h cultures of the bacterial over-expression strains were then assessed for uniform growth by OD_600_ measurement (standard error was ±2.5%) and mixed 1:1 with the prodrug dilution series in a final volume of 210 µL per well. HCT-116 monolayers were then washed twice with PBS, and 100 µL of the bacteria-prodrug mixtures were applied. The monolayers were incubated 4 h at 37 °C, 5% CO_2_, after which the bacteria were removed by washing four times in PBS and fresh 100 µL DMEM (supplemented with foetal calf serum, HEPES and 100 µg/mL chloramphenicol to kill the bacteria) was added to each monolayer. The HCT-116 cells were then allowed to recover at 37 °C, 5% CO_2_ for 48 h. Following this period, 20 µL of CellTiter 96^®^ AQueous One Solution (Promega, Madison, WI, USA) was added to each well and incubated for 2 h at 37 °C, 5% CO_2_. The OD_490_ was recorded to measure formazan levels, proportional to the number of respiring HCT-116 cells present in the culture. IC_50_ values were then calculated by comparing the OD_490_ of the challenged cells with the unchallenged cells using GraphPad Prism (Graph-pad software Inc., La Jolla, CA, USA).

## 4. Conclusions

Historically, based on their original isolation from partially purified *E. coli* extracts that contained different nitrofurazone reducing activities, NfsA has been referred to as the “major” oxygen-insensitive nitroreductase and NfsB the “minor” nitroreductase [31,32,33]. These designations, however, do not do justice to the wealth of other enzymes (represented in *E. coli* as well as other bacteria) that possess nitro-reducing capabilities; nor to the fact that NfsB exhibits comparable activity to NfsA with a number of different nitroaromatic substrates [12,34], both enzymes typically being far more active than representatives of families such as NemA, AzoR and MdaB [14]. MsuE is the first representative outside of the NfsA and NfsB families to display comparable activity with next-generation GDEPT prodrugs. This is of particular interest to us given our ongoing interest in both rationally engineering and randomly evolving superior nitroreductase variants from wild type progenitors [13]; the structural and functional novelty that an entirely new nitroreductase offers may enable unique solutions to problems of achieving superior catalytic activity. On this basis, we suggest that the results presented here merit further evaluation of MsuE in *in vivo* GDEPT models, especially in partnership with nitro-CBI-DEI prodrug.
